# The relationship between EZH2 expression and microRNA-31 in colorectal cancer and the role in evolution of the serrated pathway

**DOI:** 10.18632/oncotarget.7260

**Published:** 2016-02-08

**Authors:** Hiroyoshi Kurihara, Reo Maruyama, Kazuya Ishiguro, Shinichi Kanno, Itaru Yamamoto, Keisuke Ishigami, Kei Mitsuhashi, Hisayoshi Igarashi, Miki Ito, Tokuma Tanuma, Yasutaka Sukawa, Kenji Okita, Tadashi Hasegawa, Kohzoh Imai, Hiroyuki Yamamoto, Yasuhisa Shinomura, Katsuhiko Nosho

**Affiliations:** ^1^ Department of Gastroenterology, Rheumatology and Clinical Immunology, Sapporo Medical University School of Medicine, Sapporo, Japan; ^2^ Department of Molecular Biology, Sapporo Medical University School of Medicine, Sapporo, Japan; ^3^ The Institute of Medical Science, The University of Tokyo, Tokyo, Japan; ^4^ Department of Surgery, Surgical Oncology and Science, Sapporo Medical University School of Medicine, Sapporo, Japan; ^5^ Department of Surgical Pathology, Sapporo Medical University School of Medicine, Sapporo, Japan; ^6^ Division of Gastroenterology and Hepatology, Department of Internal Medicine, St. Marianna University School of Medicine, Kawasaki, Japan; ^7^ Department of Gastroenterology, Ikeda Municipal Hospital, Ikeda, Japan

**Keywords:** EZH2, CIMP, EGFR, ChIP, H3K27me3

## Abstract

Polycomb group protein enhancer of zeste homolog 2 (EZH2) is a methyltransferase that correlates with the regulation of invasion and metastasis and is overexpressed in human cancers such as colorectal cancer. MicroRNA-31 (miR-31) plays an oncogenic role and is associated with *BRAF* mutation and poor prognosis in colorectal cancer. EZH2 is functionally considered to suppress miR-31 expression in human cancers; however, no study has reported its relationship with colon cancer. We therefore evaluated EZH2 expression using immunohistochemistry and assessed miR-31 and epigenetic alterations using 301 colorectal carcinomas and 207 premalignant lesions. Functional analysis was performed to identify the association between EZH2 and miR-31 using cancer cell lines. In the current study, negative, weak, moderate, and strong EZH2 expressions were observed in 15%, 19%, 25%, and 41% of colorectal cancers, respectively. EZH2 was inversely associated with miR-31 (*P* < 0.0001), independent of clinicopathological and molecular features. In a multivariate stage-stratified analysis, high EZH2 expression was related to favorable prognosis (*P* = 0.0022). Regarding premalignant lesions, negative EZH2 expression was frequently detected in sessile serrated adenomas/polyps (SSA/Ps) (76%; *P* < 0.0001) compared with hyperplastic polyps, traditional serrated adenomas, and non-serrated adenomas (25–36%). Functional analysis demonstrated that the knockdown of EZH2 increased miR-31 expression. In conclusion, an inverse association was identified between EZH2 and miR-31 in colorectal cancers. Our data also showed that upregulation of EZH2 expression may be rare in SSA/Ps. These results suggest that EZH2 suppresses miR-31 in colorectal cancer and may correlate with differentiation and evolution of serrated pathway.

## INTRODUCTION

A polycomb group protein, enhancer of zeste homolog 2 (EZH2), is a methyltransferase and the core catalytic element of polycomb repressive complex 2 (PRC2), which plays a critical role in the regulation of cancer initiation, progression, invasion, metastasis, and drug resistance [1-19]. Various oncogenic transcription factors and cancer-associated non-coding RNAs regulate EZH2 expression [1-4, 6, 9, 10, 16, 17, 20, 21]. Increased EZH2 activity induces the genomewide histone H3 lysine 27 trimethylation (H3K27me3) and may act as an oncogene via the repression of tumor suppressor genes in human cancers [1-16, 20, 22-24]. In gastrointestinal cancers, EZH2 overexpression has been shown in colorectal [8, 11-14], esophageal (squamous cell carcinoma) [1, 15], gastric [10, 17, 20, 25], pancreatic [5, 26], and bile duct cancers [4]. Associations have also been reported between EZH2 overexpression and poor prognosis in esophageal [15], gastric [25], pancreatic [26], and bile duct cancers [4]. In contrast, previous studies on colorectal cancer have reported associations between EZH2 overexpression and better prognosis [11-13].

MicroRNAs constitute a class of small non-coding RNA molecules that function as post-transcriptional gene regulators and have been increasingly recognized as useful biomarkers in various human cancers [1, 2, 6, 17, 20, 27-34]. Recent evidence has shown that microRNAs can act as both oncogenes and tumor suppressors, depending on the genes they regulate [6, 17]; for example, microRNA-31 (miR-31) is reportedly deregulated in human cancers [1, 2, 6, 16, 27, 28, 30, 35], and provides oncogenic potential in colorectal cancer [27-29, 31, 36]. Using microRNA array analysis, we recently identified that miR-31 expression was significantly upregulated in *BRAF*-mutated colorectal cancer compared with wild-type colorectal cancer [28]. Moreover, associations were identified between miR-31 expression and poor prognosis in colorectal cancer [28]. We also reported that high miR-31 expression may correlate with evolution of the serrated pathway [31, 37].

A recent study has reported that EZH2 suppresses miR-31 expression by inducing H3K27me3 on the miR-31 promoter and that the inhibition of EZH2 increased miR-31 expression in prostate cancer [2]. Furthermore, EZH2-mediated histone methylation has been shown to suppress miR-31 expression in adult T-cell leukemia [16]. With regard to melanoma, genetic and epigenetic loss of miR-31 produced a feed-forward EZH2 expression [6]. Thus, accumulating evidence indicates that EZH2 may downregulate miR-31 expression in human cancers; however, no study has reported the relationship between EZH2 and miR-31 in colorectal cancer.

We conducted this study to clarify the association of EZH2 expression with miR-31 and epigenetic alterations using a database comprising more than 500 colorectal tumors. Furthermore, we performed functional analyses to identify whether EZH2 suppressed miR-31 expression in colorectal cancers.

## RESULTS

### EZH2 expression in colorectal cancer tissue and matched normal mucosa

**Figure 1:**
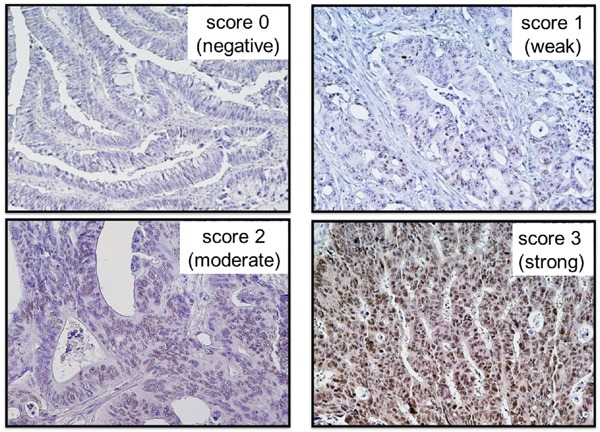
Immunohistochemical findings related to EZH2 expression in colorectal cancers Score 0 (negative), score 1 (weak), score 2 (moderate), and score 3 (strong) EZH2 expressions were observed in 15%, 19%, 25%, and 41% of the 301 colorectal cancer tissues, respectively.

Using immunohistochemistry, we assessed 310 formalin-fixed paraffin-embedded (FFPE) specimens of colorectal cancer tissues in the EZH2 expression assay (Figure 1) and successfully obtained 301 (97%) valid results. We also examined matched samples of normal colorectal mucosa (controls). EZH2 expression scores of 0 (negative), 1 (weak), 2 (moderate), and 3 (strong) were observed in 15%, 19%, 25%, and 41% of the colorectal cancer tissues, respectively (Table 1). EZH2 expression in colorectal cancer tissues was significantly higher than its expression in normal mucosa tissues (*P* < 0.0001) (Supplementary Figure 1).

**Table 1:** Clinicopathological and molecular features of 301 colorectal cancers according to EZH2 expression

Clinicopathological or molecular feature	Total N	EZH2 expression				*p*
		score 0 (negative)	score 1 (weak)	score 2 (moderate)	score 3 (strong)	
All cases	301	44	58	76	123	
Gender						
Male	166 (55%)	27 (61%)	39 (67%)	41 (54%)	59 (48%)	0.078
Female	135 (45%)	17 (39%)	19 (33%)	35 (46%)	64 (52%)	
Age (mean ± SD)	65.5 ± 12.3	67.3 ± 11.3	65.3 ± 11.8	65.7 ± 11.4	64.8 ± 13.4	0.71
Tumor size (mm) (mean ± SD)	52.6 ± 25.0	58.5 ± 31.7	53.3 ± 24.9	51.5 ± 22.4	50.9 ± 24.0	0.37
Year of diagnosis						
Prior to 2003	155 (52%)	29 (66%)	32 (55%)	39 (51%)	55 (45%)	0.16
2004-2006	79 (26%)	6 (14%)	17 (29%)	21 (28%)	35 (28%)	
Posterior to 2007	67 (22%)	9 (20%)	9 (16%)	16 (21%)	33 (27%)	
Tumor location						
Rectum and Distal colon (splenic flexure to sigmoid)	213 (71%)	30 (68%)	36 (62%)	53 (70%)	94 (76%)	0.24
Proximal colon (cecum to transverse)	88 (29%)	14 (32%)	22 (38%)	23 (30%)	29 (24%)	
Tumor differentiation						
Well to Moderate	270 (90%)	36 (82%)	52 (90%)	72 (95%)	110 (89%)	0.17
Poor	31 (10%)	8 (18%)	6 (10%)	4 (5.3%)	13 (11%)	
Disease stage						
I	8 (2.7%)	0 (0%)	1 (1.7%)	2 (2.6%)	5 (4.1%)	0.30
IIA	34 (11%)	7 (16%)	5 (8.6%)	9 (12%)	13 (11%)	
IIB	22 (7.3%)	6 (14%)	3 (5.2%)	5 (6.6%)	8 (6.5%)	
IIIA	28 (9.3%)	2 (4.6%)	6 (10%)	3 (4.0%)	17 (14%)	
IIIB	103 (34%)	13 (30%)	21 (36%)	24 (32%)	45 (37%)	
IIIC	56 (19%)	7 (16%)	15 (26%)	16 (21%)	18 (15%)	
IV	50 (17%)	9 (20%)	7 (12%)	17 (22%)	17 (14%)	
*BRAF* mutation						
Wild-type	288 (96%)	42 (95%)	52 (90%)	74 (97%)	120 (98%)	0.13
Mutant	13 (4.3%)	2 (4.6%)	6 (10%)	2 (2.6%)	3 (2.4%)	
*KRAS* mutation						
Wild-type	204 (68%)	23 (52%)	42 (72%)	49 (64%)	90 (73%)	0.066
Mutant	97 (32%)	21 (48%)	16 (28%)	27 (36%)	33 (27%)	
*PIK3CA* mutation						
Wild-type	267 (89%)	40 (91%)	49 (84%)	67 (88%)	111 (90%)	0.68
Mutant	34 (11%)	4 (9.1%)	9 (16%)	9 (12%)	12 (9.8%)	
CIMP status						
CIMP-low/zero	283 (94%)	39 (89%)	54 (93%)	75 (99%)	115 (94%)	0.10
CIMP-high	18 (6.0%)	5 (11%)	4 (6.9%)	1 (1.3%)	8 (6.5%)	
MSI status						
MSS/MSI-low	279 (93%)	40 (91%)	55 (95%)	72 (95%)	112 (91%)	0.66
MSI-high	22 (7.3%)	4 (9.1%)	3 (5.2%)	4 (5.3%)	11 (8.9%)	
miR-31 expression						
Low expression (Q1, Q2 and Q3)	226 (75%)	20 (45%)	36 (62%)	60 (79%)	110 (89%)	< 0.0001
High expression (Q4)	75 (25%)	24 (55%)	22 (38%)	16 (21%)	13 (11%)	

Percentage (%) indicates the proportion of cases with a specific clinicopathological or molecular feature within a given category (score 0, score 1, score 2, score 3) of EZH2 expression by immunohistochemistry. *P*-values were calculated by analysis of variance for age and tumor size and by a chi-square test or Fisher's exact test for all other variables. To account for multiple hypothesis testing in associations between EZH2 expression and other 13 covariates, the *P*-value for significance was adjusted by Bonferroni correction to *P* = 0.0038 (= 0.05/13).

CIMP, CpG island methylator phenotype; MSI, microsatellite instability; MSS, microsatellite stable; miR-31, microRNA-31; SD, standard deviation.

### MicoroRNA-31 expression in colorectal cancer

The distributions of miR-31 expression in the 301 colorectal cancers were as follows: mean 55.4; median 10.7; standard deviation (SD) 211; range 0.11–2108; interquartile range 3.9–32.2 (Supplementary Figure 2). Cases with miR-31 expression were divided into quartiles Q1 (<3.9), Q2 (3.9–10.6), Q3 (10.7–32.1), and Q4 (≥32.2) for further analysis.

### The association between EZH2 expression and clinical, pathological and molecular characteristics in colorectal cancer

**Figure 2:**
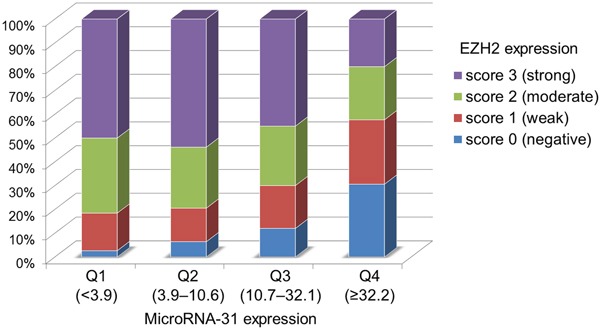
The association between EZH2 expression and microRNA-31 expression in 301 colorectal cancers EZH2 expression levels were inversely associated with microRNA-31 expressions in colorectal cancers (*P* < 0.0001).

Table 1 summarises the clinical features of all 301 patients with colorectal cancer according to EZH2 expression. No significant association existed between EZH2 expression and the clinical or pathological characteristics such as gender, age, tumor size, year of diagnosis, tumor location, tumor differentiation and disease stage; *BRAF*, *KRAS*, and *PIK3CA* mutations; and the MSI (microsatellite instability) and CpG island methylator phenotype (CIMP) status (Table 1). CIMP-specific promoter methylation (*CACNA1G*, *IGF2*, *MLH1*, or *RUNX3*) was not associated with EZH2 expression (data not shown). However, EZH2 expression was inversely associated with miR-31 expression (*P* < 0.0001) (Table 1 and Figure 2).

### EZH2 expression and patient survival

**Figure 3:**
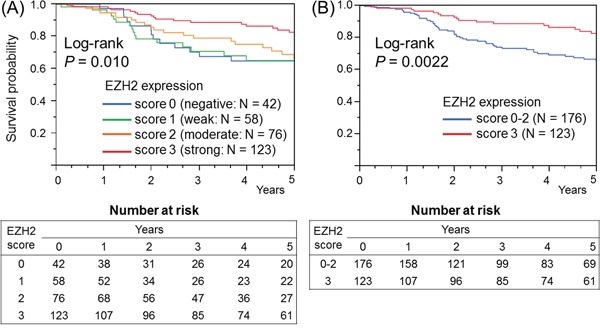
Kaplan–Meier survival curves for colorectal cancer (stages I-IV) according to the EZH2 expression level **A.** In terms of cancer-specific survival, significantly lower mortality was observed in patients with high EZH2 expression than in those with low EZH2 expression (log-rank test: *P* = 0.010). **B.** In terms of cancer-specific survival, significantly lower mortality was observed in patients with high expression (score 3) than in those with low expression (score 0–2) (log-rank test: *P* = 0.0022).

The influence of EZH2 expression on clinical outcomes was assessed in 299 patients with colorectal cancer (stages I–IV). During follow-up among eligible patients, 100 patients died, of which 81 deaths were attributed to colorectal cancer. The median follow-up time for censored patients was 4.4 years. Kaplan–Meier analysis was performed using categorical variables (score 0, score 1, score 2, or score 3). In terms of cancer-specific survival, significantly lower mortality was observed in patients with high EZH2 expression (log-rank test: *P* = 0.010) than in those with low EZH2 expression (Figure 3A).

We made a dichotomous expression variable for EZH2, defining a score of 3 as high expression and scores of 0–2 as low expression. In terms of cancer-specific survival, significantly lower mortality was observed in patients with high expression (log-rank test: *P* = 0.0022) than in those with low expression (Figure 3B). However, no significant differences were observed between the high and low expression groups when we defined scores of 1–3 as high expression and score of 0 as low expression (log-rank test: *P* = 0.69) or when we defined scores of 2–3 as high expression and scores of 0–1 as low expression (*P* = 0.075).

In univariate Cox regression analysis for cancer-specific survival, significantly lower mortality was observed in the high expression group (score 3) compared with the low expression group (score 0–2) [hazard ratio (HR): 0.48; 95% confidence interval (CI): 0.29–0.77; *P* = 0.0018] (Supplementary Table 1). Similarly, in comparison with the low expression group, an independent association with a favorable prognosis was observed in the high expression group in both stage-stratified (HR: 0.51; 95% CI: 0.29−0.77; *P* = 0.0022) and multivariate analyses (HR: 0.46; 95% CI: 0.27−0.76; *P* = 0.0022) (Supplementary Table 1).

### Multivariate logistic regression analysis in high EZH2 expression group

Considering potential confounders and cause-and-effect sequences, we performed multivariate logistic regression analyses to assess the relationships with EZH2 expression. In the final model, high EZH2 expression (score 3) was inversely associated with high miR-31 expression (Q4) [odds ratio (OR): 0.22; 95% CI: 0.11–0.42; *P* < 0.0001] (Table 2).

**Table 2:** Multivariate logistic regression analysis of EZH2 expression in colorectal cancers

Variables in the final model for EZH2 expression (as an outcome variable) [High expression group (score 3) vs. Low expression group (score 0-2)]	Adjusted odds ratio (95% CI)	*P*
High microRNA-31 expression (vs. Low expression)	0.22 (0.11-0.42)	< 0.0001
Female gender (vs. Male)	1.78 (1.08-2.98)	0.025

A multivariate logistic regression analysis assessing the relationships with EZH2 expression status initially included gender, age, tumor size, year of diagnosis, tumor location, tumor differentiation, disease stage, CIMP status, MSI status, mutations of BRAF, KRAS and PIK3CA, and microRNA-31 expression, considering potential confounding and causal relationships. For multiple hypothesis testing, the P-value for significance was adjusted by Bonferroni correction to 0.0038 (= 0.05/13).

CI, confidence interval; CIMP, CpG island methylator phenotype; MSI, microsatellite instability.

### Association of EZH2 expression with clinicopathological and molecular features in premalignant colorectal lesions

We assessed 215 FFPE tissue specimens from premalignant colorectal lesions using immunohistochemistry (Supplementary Figure 3) and successfully obtained 207 (96%) valid results. Table 3 shows the clinicopathological and molecular features, including EZH2 expression in serrated lesions and non-serrated adenomas. Negative EZH2 expression (score 0) was frequently detected in sessile serrated adenomas/polyps (SSA/Ps) (76%) compared with hyperplastic polyps (HPs) (36%), traditional serrated adenomas (TSAs) (25%), and non-serrated adenomas (36%). EZH2 expression [score 2 (moderate) or score 3 (strong)] was not detected in any SSA/Ps.

**Table 3:** Clinicopathological and molecular features of 152 serrated lesions and 55 non-serrated adenomas

Clinicopathological or molecular feature	Total N	Histological type				*p*
		Hyperplastic polyp (HP)	Sessile serrated adenoma/polyp (SSA/P)	Traditional serrated adenoma (TSA)	Non-serrated adenoma	
All cases	207	50	51	51	55	
Gender						
Male	124 (60%)	36 (72%)	23 (45%)	26 (51%)	39 (71%)	0.0065
Female	83 (40%)	14 (28%)	28 (55%)	25 (49%)	16 (29%)	
Age (mean ± SD)	61.7 ± 10.8	58.9 ± 11.6	57.4 ± 10.8	65.1 ± 11.5	65.3 ± 8.7	< 0.0001
Tumor size (mm) (mean ± SD)	11.0 ± 5.3	9.0 ± 3.7	13.5 ± 6.7	9.9 ± 4.7	11.4 ± 5.4	0.0002
Tumor location						
Rectum and Distal colon	98 (47%)	24 (48%)	5 (9.8%)	38 (75%)	31 (56%)	< 0.0001
Proximal colon	109 (53%)	26 (52%)	46 (90%)	13 (25%)	24 (44%)	
*BRAF* mutation						
Wild-type	107 (52%)	27 (54%)	7 (14%)	19 (37%)	54 (98%)	< 0.0001
Mutant	100 (48%)	23 (46%)	44 (86%)	32 (63%)	1 (1.8%)	
CIMP status						
CIMP-low/zero	174 (84%)	46 (92%)	32 (63%)	41 (80%)	55 (100%)	< 0.0001
CIMP-high	33 (16%)	4 (8.0%)	19 (37%)	10 (20%)	0 (0%)	
MicroRNA-31						
Low expression (Q1-3)	155 (75%)	39 (78%)	40 (78%)	27 (53%)	49 (89%)	< 0.0001
High expression (Q4)	52 (25%)	11 (22%)	11 (22%)	24 (47%)	6 (11%)	
EZH2 expression						
Score 0 (negative)	90 (43%)	18 (36%)	39 (76%)	13 (25%)	20 (36%)	< 0.0001
Score 1 (weak)	83 (40%)	20 (40%)	12 (24%)	23 (45%)	28 (51%)	
Score 2 (moderate)	34 (16%)	12 (24%)	0 (0%)	15 (29%)	7 (13%)	
Score 3 (strong)	0 (0%)	0 (0%)	0 (0%)	0 (0%)	0 (0%)	

*P*-values were calculated by analysis of variance for age and tumor size, and by a chi-square test for all other variables. CIMP, CpG island methylator phenotype; HP, hyperplastic polyp; SSA/P, sessile serrated adenoma/polyp; SD, standard deviation; TSA, traditional serrated adenoma.

### Knockdown of EZH2 increases miR-31 expression in colon cancer cell lines

**Figure 4:**
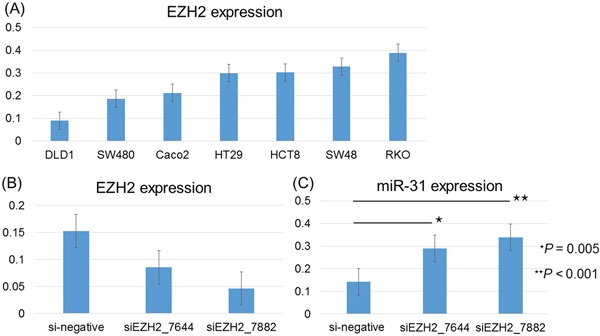
EZH2 knockdown caused microRNA-31 (miR-31) overexpression on quantitative RT-PCR **A.** EZH2 expression is shown according to each of the 7 colorectal cancer cell lines studied. Error bars represent the standard deviations. **B.** The siRNA-mediated knockdown of EZH2 caused a significant reduction in EZH2 expression in RKO cells transfected with EZH2 siRNAs (siEZH2_7644 and siEZH2_7882). **C.** There was a considerable increase in miR-31 expression in RKO cells transfected with EZH2 siRNAs (siEZH2_7644 and siEZH2_7882). The *P*-value was analyzed using paired T-test.

To examine whether EZH2 suppressed miR-31 expression, we knocked down EZH2 mRNA by siRNAs in colon cancer cell lines and measured the resulting miR-31 expression. Figure 4A shows the expression level of EZH2 in 7 colon cancer cell lines using quantitative reverse transcription-PCR (qRT-PCR) (Figure 4A). Our data demonstrated that in RKO cells, EZH2 expression was successfully downregulated by approximately 30% and 56% when transfecting two different EZH2 siRNAs (siEZH2_7644 and siEZH2_7882, respectively) (Figure 4B). Moreover, we found that there was a considerable increase in miR-31 expression in RKO cells transfected with EZH2 siRNAs (Figure 4C). Similar results were observed in HT29 and SW480 cells (Supplementary Figure 4).

### Promoter region of miR-31 is marked by histone H3 lysine 27 trimethylation (H3K27me3)

To clarify the functional link between EZH2 and miR-31 expression, we examined H3K27me3 levels around the promoter region of miR-31 by performing chromatin immunoprecipitation (ChIP) assay. We observed that H3K27me3 was steadily enriched at the promoter region of miR-31 in RKO cells, and the H3K27me3 levels were decreased after knockdown of EZH2 (Supplementary Figure 5). Taken together with increased expression of miR-31 after EZH2 knockdown (Figure 4C), these results suggest the expression of miR-31 is suppressed by EZH2 through H3K27me3 in RKO cells.

## DISCUSSION

We performed this study to identify the association of EZH2 expression with molecular alterations in colorectal tumors. In a database comprising 301 patients with colorectal cancer, high EZH2 expression was inversely associated with miR-31 expression, independent of clinicopathological and molecular features. Our data also showed that high EZH2 expression was a favorable prognostic factor in colorectal cancer. Regarding premalignant lesions, negative EZH2 expression was higher in SSA/Ps than in HPs, TSAs or non-serrated adenomas. In functional analysis, we showed that the siRNA knockdown of EZH2 increased miR-31 expression in colon cancer cell lines.

EZH2 reportedly downregulates miR-31 expression in human cancers such as prostate cancer [2] and adult T-cell leukemia [16]. However, no study has reported the potential role of EZH2 in the regulation of miR-31 expression in colorectal cancer. The present multivariate analysis showed that high EZH2 expression was inversely associated with miR-31 expression in colorectal cancer, whereas functional analysis showed that EZH2 knockdown increased miR-31 expression in colon cancer cell lines. Recent studies that used colon cancer cell lines have reported that EZH2 expression was inversely associated with microRNA-506 [38], microRNA-26a, and let-7b [39]; these microRNAs downregulated EZH2 expression by directly targeting 3′-UTR. Conversely, EZH2 has been reported to suppress miR-31 expression by inducing H3K27me3 on the miR-31 promoter in prostate cancer. Therefore, to determine if EZH2 is involved in the regulation of miR-31 expression through histone H3K27me3, we examined H3K27me3 levels around the transcription start site on the miR-31 promoter by performing ChIP assay and found that EZH2-mediated histone methylation downregulates miR-31 expression in colon cancer. To the best of our knowledge, this is the first report describing the downregulation of miR-31 by EZH2 in colorectal cancer. Recently, we also reported that high miR-31 expression was associated with shorter progression-free survival in patients with colorectal cancer treated using anti-epidermal growth factor receptor (EGFR) therapy [27]. Therefore, because of the relationship with miR-31, EZH2 may represent a new prognostic biomarker for molecular targeted therapies and can provide a promising therapeutic target in patients with colorectal cancer.

With regard to the association between EZH2 expression and outcomes in patients with colorectal cancer, previous studies have reported that EZH2 overexpression was associated with a favorable prognosis [11-13]. Our data also showed that high EZH2 expression was associated with favorable survival using multivariate stage-stratified Cox analysis. These results are reasonable because we recently reported that high miR-31 expression, which is inversely correlated with EZH2 expression, is an unfavorable prognostic factor in patients with colorectal cancer [28]. We summarized the association of EZH2 expression with miR-31 and cancer-specific survival in Supplementary Figure 6. Our current study had some important limitations, particularly because of its cross-sectional nature and unknown bias (i.e. selection bias). Nevertheless, our multivariate regression analysis was adjusted for potential confounders, including clinical and molecular features, and we were able to show that high EZH2 expression was inversely associated with miR-31 expression and associated with better prognosis.

CIMP is a distinct form of epigenomic instability that causes most cases of sporadic MSI-high colorectal cancers through the epigenetic inactivation of *MLH1* [30, 31, 40-44]. *RUNX3* belongs to the *RUNX* family of genes, which has been reported to be the single best marker for diagnosing CIMP-high status [41]. Previous studies have reported that EZH2 downregulates RUNX3 by inducing histone H3 methylation in various human cancers such as gastric [10], bile duct [4], and pancreatic cancers [5]. With regard to colorectal cancer, Kodach et al. reported, that despite higher levels of EZH2 and lower levels of RUNX3, no inverse correlation was present between EZH2 and RUNX3 [9]. Consistent with those results, when using 4 CIMP-specific promoters (*CACNA1G*, *IGF2*, *MLH1*, and *RUNX3*), we showed that neither *RUNX3* methylation nor CIMP-high status was associated with EZH2 expression. These results suggest that EZH2 overexpression may not correlate with the epigenetic silencing of CIMP-specific promoters by histone H3 methylation in colorectal cancer.

Premalignant colorectal neoplasms appear to be important precursor lesions in the pathogenesis of colorectal cancer. In particular, the serrated neoplasia pathway has attracted considerable attention as an alternative pathway of colorectal cancer development, and serrated lesions exhibit unique clinicopathological and molecular features [45-48]. Both SSA/Ps and TSAs are recognized premalignant lesions, but SSA/Ps are the principle serrated precursors of colorectal cancer [47-50]. Because there are many clinicopathological and molecular similarities between SSA/Ps and CIMP-high colorectal cancers, including proximal tumor location, *BRAF* mutation, and *MLH1* methylation, SSA/Ps are hypothesized to be precursor lesions that develop into CIMP-high colorectal cancers [31, 42, 46, 50, 51].

We recently reported that high miR-31 expression was more pronounced in SSA/Ps with cytological dysplasia than in other SSA/Ps, but we did not find a significant difference between TSAs with high-grade dysplasia and other TSAs [31]. These data imply that miR-31 correlate with the progression of SSA/Ps. However, our current data revealed that EZH2 expression (moderate or strong) was not detected in any SSA/Ps and that negative EZH2 expression was higher in SSA/Ps than in HPs, TSAs, and non-serrated adenomas. In addition, we demonstrated that the knockdown of EZH2 by siRNAs increased miR-31 expression in colon cancer cell lines, suggesting that negative EZH2 expression causes miR-31 upregulation in the progression of SSA/Ps. Therefore, EZH2 may be a key molecule in the differentiation and evolution of serrated lesions, and microvesicular HPs without EZH2 expression may progress to SSA/Ps. Future independent studies are required to clarify the role of EZH2 expression in the serrated pathway.

In conclusion, we identified an inverse association between the expressions of EZH2 and miR-31 in colorectal cancer and that the upregulation of EZH2 expression may be a rare event in SSA/Ps. Hence, we suggest that EZH2 suppresses miR-31 expression in colorectal cancer and may correlate with differentiation and evolution of the serrated pathway. These findings improve our understanding of the mechanism of colorectal tumorigenesis and have the potential to significantly affect clinical and translational research on colorectal cancer.

## MATERIALS AND METHODS

### Patients and tissue specimens

Formalin-fixed paraffin-embedded (FFPE) tissues of 310 colorectal cancers (stages I–IV), 158 serrated lesions and 57 non-serrated adenomas (i.e. tubular or tubulovillous adenomas) of patients who underwent endoscopic resection or other surgical treatment at Sapporo Medical University Hospital and Keiyukai Sapporo Hospital between 1999 and 2014 were collected. To avoid selection bias as much as possible, we consecutively collected FFPE tissue specimens of colorectal cancers, serrated lesions, and non-serrated adenomas.

The criterion for diagnosis of colorectal cancer was invasion of malignant cells beyond the muscularis mucosa. Intramucosal carcinoma and carcinoma in situ were classified as adenoma. Colorectal tumors were classified by location as follows: the proximal colon (cecum, ascending, and transverse colon), distal colon (splenic flexure, descending, and sigmoid colon) and rectum. The patients were followed until death or December 2014, whichever came first.

Serrated lesions [hyperplastic polyps (HPs) (N = 54), sessile serrated adenomas/polyps (SSA/Ps) (N = 53) and traditional serrated adenomas (TSAs) (N = 51)] were classified on the basis of the current World Health Organization (WHO) criteria [52]. All HPs were found to be microvesicular. Informed consent was obtained from all the patients before specimen collection. This study was approved by the respective institutional review boards of the participating institutions.

### RNA extraction and qRT-PCR of microRNA-31

Total RNA was extracted from FFPE tissues using the miRNeasy FFPE Kit (Qiagen, Valencia, CA, USA) [28]. MicroRNA-31 (miR-31)-5p expression was analyzed by qRT-PCR using TaqMan MicroRNA Reverse Transcription Kit (Applied Biosystems, Foster City, CA, USA) and TaqMan microRNA Assays (Applied Biosystems) as described previously [28]. U6 snRNA (RNU6B; Applied Biosystems) served as an endogenous control. miR-31 expression was calculated using the equation 2^−ΔCT^, where ΔC_T_ = (C_T_ miR-31 − C_T_ U6). To calculate the relative expression of miR-31 in each colorectal cancer, 2^−ΔCT^ of cancer tissue was divided by 2^−ΔCT^ of normal tissue, as described previously [28].

### DNA extraction and pyrosequencing of *KRAS, BRAF*, and *PIK3CA* mutations and MSI analysis

Genomic DNA was extracted from FFPE tissues of colorectal tumors using QIAamp DNA FFPE Tissue Kit (Qiagen) [28]. Using extracted genomic DNA, PCR and targeted pyrosequencing were performed for *KRAS* (codon 12 or 13) [27], *BRAF* (codon 600) [28], and *PIK3CA* (exon 9 or 20) [40]. MSI analysis was performed as described previously [28].

### Sodium bisulfite treatment and real-time PCR (MethyLight) to measure promoter methylations of *CACNA1G*, *IGF2*, *MLH1*, and *RUNX3*

Bisulfite modification of genomic DNA was performed using a BisulFlash™ DNA Modification Kit (Epigentek, Brooklyn, NY, USA) [28]. We quantified DNA methylation in 4 CIMP-specific promoters (*CACNA1G, IGF2, MLH1*, and *RUNX3*) by real-time PCR (MethyLight), as described previously [30, 31, 41]. CIMP-high was defined as the presence of three/four or more methylated promoters and CIMP low/zero as zero/four to two/four methylated promoters [31].

### Immunohistochemistry for EZH2 expression

Immunohistochemistry was performed on 5 μm FFPE sections. Sections were autoclave-pretreated in target retrieval solution (Dako Cytomation, Carpinteria, CA, USA). Endogenous peroxidase activity was blocked using 3% hydrogen peroxide, and the sections were incubated overnight at 4°C with anti-EZH2 antibody (#612667, BD Biosciences, San Jose, CA, USA) at a dilution of 1:100. A subsequent reaction was performed using a horseradish peroxidase enzyme-labeled polymer of the EnVision™ Plus detection system (Dako). When a positive reaction was visualized using a diaminobenzidine (DAB) solution, counterstaining was performed using Mayer's hematoxylin. Five random high-power fields were evaluated per lesion to determine the mean nuclear positivity, which was categorized as follows: score 0 (negative, <5%), score 1 (weak, 5%–39%), score 2 (moderate, 40%–79%), or score 3 (strong, ≥80%). EZH2 expression was visually interpreted by H.K., who was unaware of the other data. For the agreement study on EZH2 expression, 147 randomly selected cases were examined by a second pathologist (by K.N.), who was also unaware of the other data. The concordance between the two pathologists (*P* < 0.0001) was 0.87 (κ = 0.74), indicating substantial agreement.

### Colon cancer cell line and small interfering RNA (siRNA) transfection

In this study, we used 7 colon cancer cell lines (Caco2, DLD1, HCT8, HT29, RKO, SW48, and SW480). Twenty-four hours after plating, the cells were transfected with either negative control siRNA (Sigma-Aldrich, St. Louis, MO, USA) or two different EZH2-targeting siRNAs (Sigma-Aldrich; siRNA ID SASI_Hs01_00147882, SASI_Hs02_00337644) using the Lipofectamine^®^ RNAiMax (Invitrogen by Life Technologies, Carlsbad, CA, USA). Fourty-eight hours after transfection, the cells were harvested for qRT-PCR.

### RNA isolation and quantitative RT-PCR

Total RNA was extracted from cell pellets using the TRIzol^®^ reagent (Invitrogen by Life Technologies) and reverse transcribed to cDNA with the PrimeScript™ RT Reagent Kit (Takara Bio Inc., Kusatsu, Japan). Quantitative RT-PCR was performed using the TaqMan^®^ universal master mix with specific primers and a probe set for EZH2 (Applied Biosystems; TaqMan Gene Expression Assay). Actin-beta (ACTB) expression was used to normalize for variance. All genes were tested in triplicates.

### Chromatin immunoprecipitation (ChIP)-PCR

ChIP was performed as described previously [35] with minor modifications. Briefly, 1 × 10^6^ cells were fixed in 1% formaldehyde for 10 min at room temperature, rinsed in glycine, then washed in cold phosphate buffered saline (PBS) twice. The cells were re-suspended in SDS lysis buffer and were sonicated using a Covaris S2 device (Covaris Inc., Woburn, MA, USA), following the manufacturers' instructions. Sheared chromatin was immunoprecipitated for more than eight hours at 4°C using 2 μg of anti-H3K27me3 antibody (#9733; Cell Signaling Technology, Danvers, MA, USA). Before adding antibodies, 10 μl of sheared chromatin was saved as input DNA sample. After washing, elution and reversal of the cross-links, DNA was purified using Agencourt AMPure XP^®^ (Agencourt Biosciences, Beverly, MA, USA), according to the product manual. The purified DNA was amplified by real-time quantitative PCR with SYBR^®^ Select Master Mix (Life Technologies) and 7500 fast real-time PCR system (Life Technologies) and was analysed for enrichment. The sequences of the ChIP primers are provided in Supplementary Table 2.

### Statistical analysis

JMP (version 10) and SAS (version 9) software programs were used for statistical analyses (SAS Institute, Cary, NC, USA). All *P*-values were two-sided. Univariate analyses were performed to investigate clinicopathological and molecular characteristics according to the EZH2 expression level; a chi-square test or Fisher's exact test was used for categorical data, while analysis of variance was used to compare the mean patient age and tumor size. To account for multiple hypothesis testing in associations between EZH2 expression and other 13 covariates, the *P*-value for significance was adjusted by Bonferroni correction to *P* = 0.0038 (= 0.05/13).

In survival analysis, the Kaplan–Meier method and log-rank test were used to assess the survival time distribution. Cox proportional hazards regression models were used to compute mortality HRs according to the EZH2 expression status. Stratification by the tumor-node-metastasis (TNM) disease stage (I, IIA, IIB, IIIA, IIIB, IIIC and IV) was performed using the “strata” option in the SAS “proc phreg” command. The multivariate, stage-stratified Cox model included the EZH2 expression variable stratified by gender (male vs. female), age at diagnosis (continuous), tumor size (continuous), year of diagnosis (continuous), tumor location (proximal colon vs. distal colon and rectum), tumor differentiation (well to moderate vs. poor), MSI status (MSI-high vs. MSS/MSI-low), CIMP status (CIMP-high vs. CIMP-low/zero), mutations of *BRAF*, *KRAS* and *PIK3CA* (present vs. absent), and miR-31 (high expression vs. low expression). A backward elimination was performed with a threshold of *P* = 0.10, to avoid overfitting.

A multivariate logistic regression analysis assessing the relationships with EZH2 expression status initially included gender, age, tumor size, year of diagnosis, tumor location, tumor differentiation, disease stage, MSI, CIMP, mutations of *BRAF*, *KRAS* and *PIK3CA*, and miR-31, considering potential confounding and causal relationships. For multiple hypothesis testing, the *P*-value for significance was adjusted by Bonferroni correction to 0.0038 (= 0.05/13).

## SUPPLEMENTARY FIGURES AND TABLE



## Abbreviations

ChIP: chromatin immunoprecipitation
CI: confidence interval
CIMP: CpG island methylator phenotype
EGFR: epidermal growth factor receptor
EZH2: enhancer of zeste homolog 2
FFPE: formalin-fixed paraffin-embedded
HP: hyperplastic polyp
HR: hazard ratio
miR-31: microRNA-31
MSI: microsatellite instability
MSS: microsatellite stable
PRC2: polycomb repressive complex 2
qRT-PCR: quantitative reverse transcription-PCR
SD: standard deviation
siRNA: small interfering RNA
SSA/P: sessile serrated adenoma/polyp
TSA: traditional serrated adenoma
H3K27me3: histone H3 lysine 27 trimethylation
WHO: World Health Organization.
