# Supplementary material for: The relationship between EZH2 expression and microRNA-31 in colorectal cancer and the role in evolution of the serrated pathway
Source: Oncotarget. 2016 Feb 8;7(11):12704–17. doi: 10.18632/oncotarget.7260 (PMC4914316; doi:10.18632/oncotarget.7260)
Supplement: Supplementary file 1 [file oncotarget-07-12704-s001.pdf]

SUPPLEMENTARY FIGURES AND TABLES

EZH2 expression in colorectal cancer tissue and matched normal mucosa

	Score 0 (Negative)	Score 1 (Weak)	Score 2 (Moderate)	Score 3 (Strong)	Total	*P*
Colorectal cancer	3	10	14	23	50	< 0.0001
Matched normal mucosa	42	7	1	0	50	

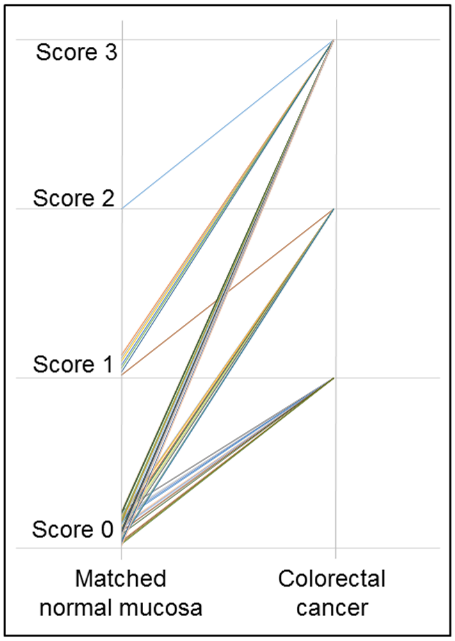

Supplementary Figure 1: EZH2 expression in 50 colorectal cancer tissues and matched normal mucosa tissues. EZH2 expression in colorectal cancer tissues was significantly higher than that in normal mucosa ($P < 0.0001$).

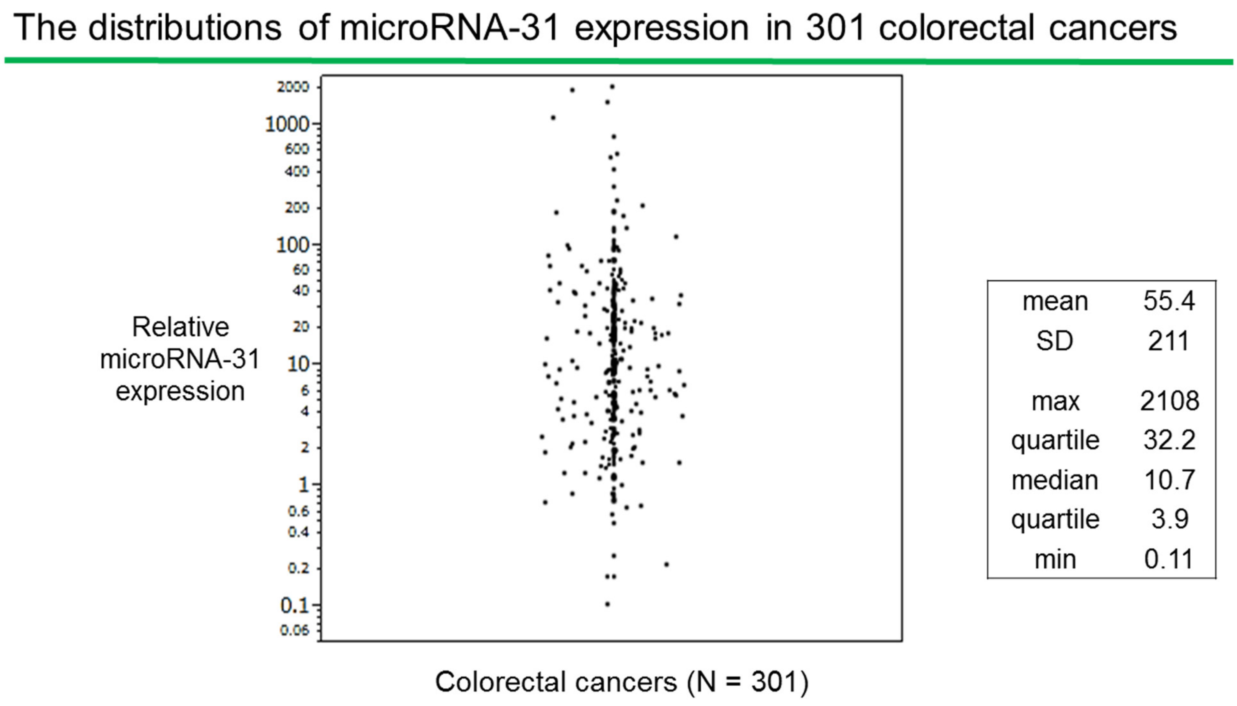

Supplementary Figure 2: The distributions of microRNA-31 expression in 301 colorectal cancers. Mean 55.4; median 10.7; standard deviation (SD) 211; range 0.11–2108; interquartile range 3.9–32.2.

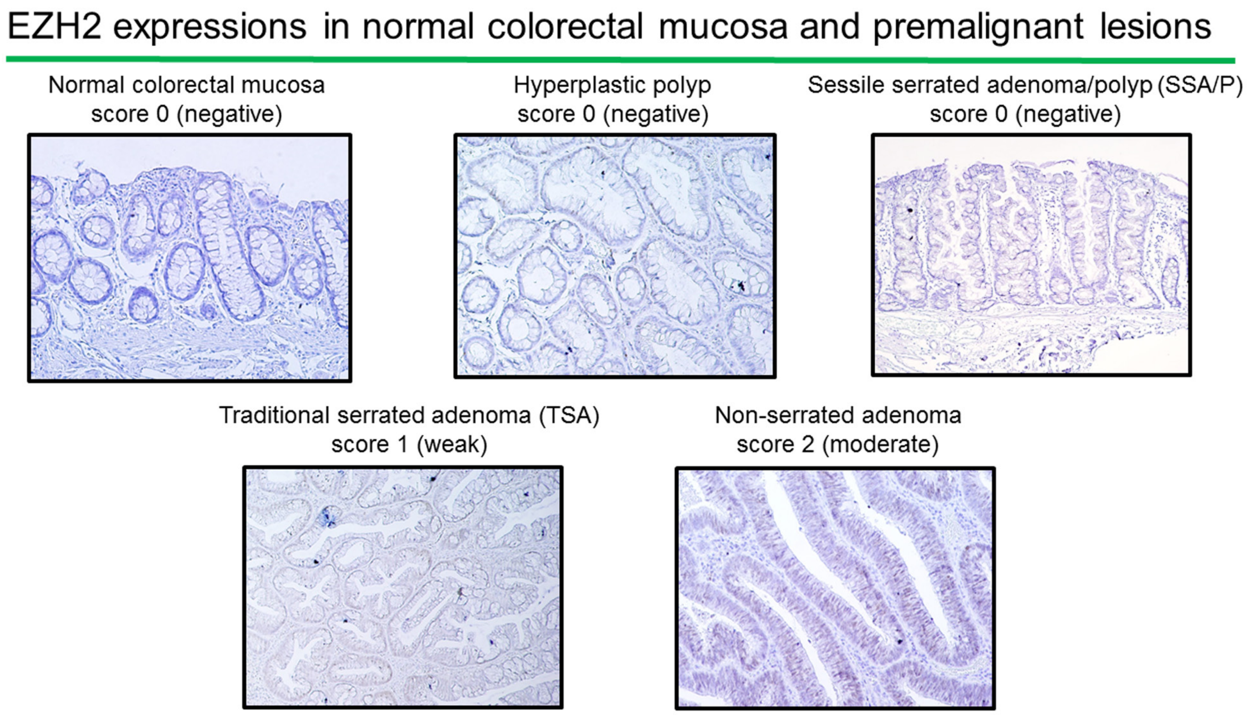

Supplementary Figure 3: EZH2 expressions in normal colorectal mucosa and premalignant lesions. Negative EZH2 expression (score 0) was frequently detected in SSA/Ps (76%), compared with HPs (36%), TSAs (25%), and non-serrated adenomas (36%). Moderate (score 2) or strong (score 3) EZH2 expression was not detected in any SSA/P. HP, hyperplastic polyp; SSA/P, sessile serrated adenoma/polyp; TSA, traditional serrated adenoma.

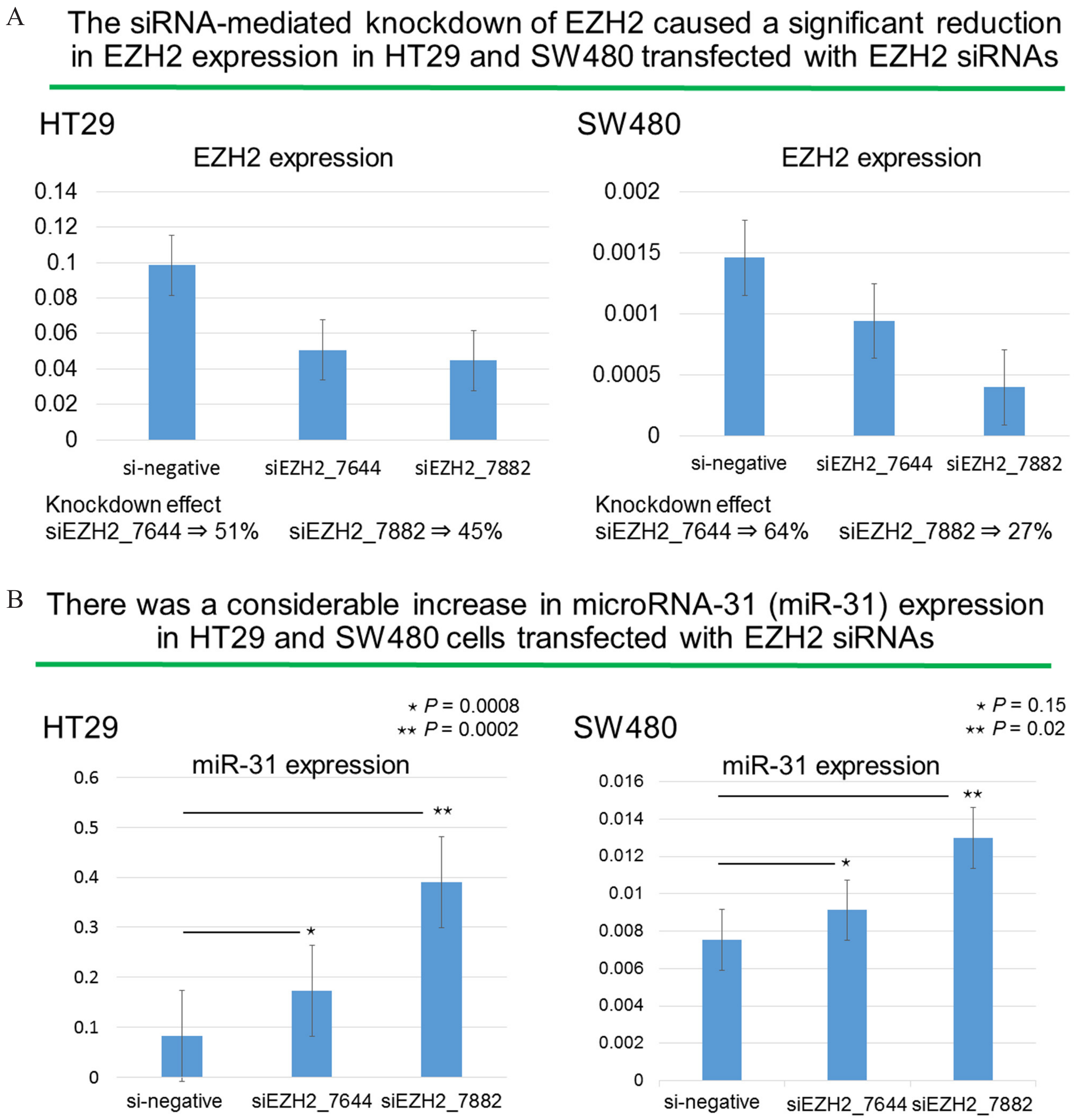

Supplementary Figure 4: EZH2 knockdown caused microRNA-31 overexpression on quantitative RT-PCR. A. The siRNA-mediated knockdown of EZH2 caused a significant reduction in EZH2 expression in HT29 and SW480 cells transfected with EZH2 siRNAs (siEZH2_7644 and siEZH2_7882). **B.** There was a considerable increase in miR-31 expression in HT29 and SW480 cells transfected with EZH2 siRNAs (siEZH2_7644 and siEZH2_7882). The *P*-value was analyzed using paired T-test.

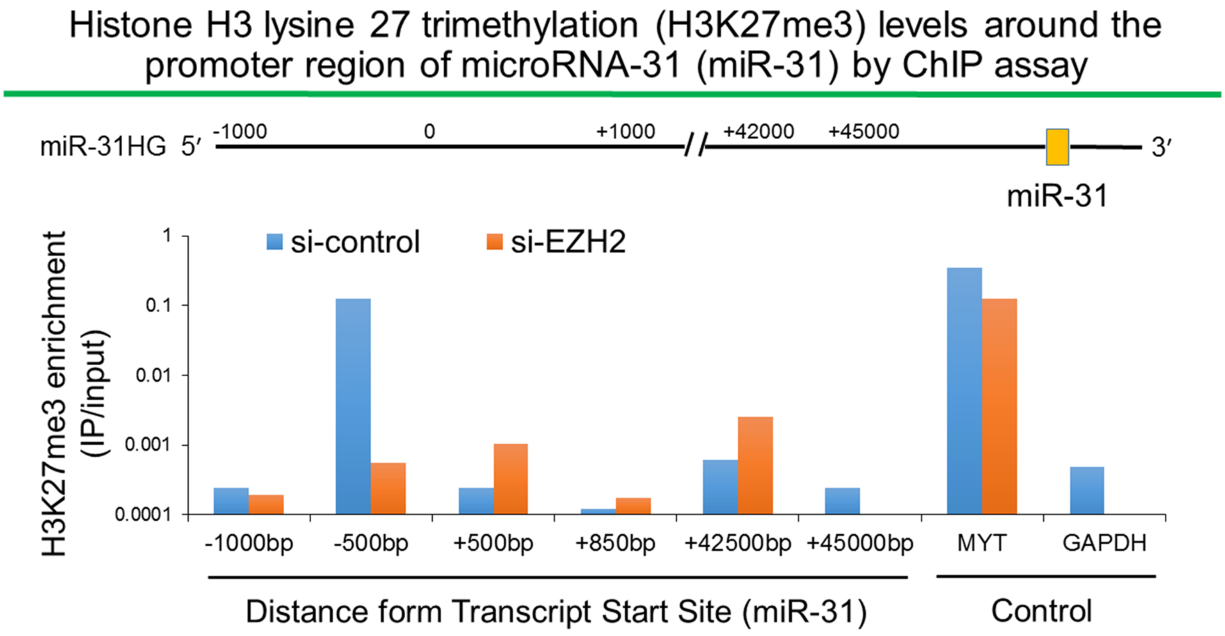

Supplementary Figure 5: H3K27me3 levels around the promoter region of microRNA-31 by performing ChIP assay. H3K27me3 was steadily enriched at the promoter region of microRNA-31 in RKO cells. The H3K27me3 levels were decreased after knockdown of EZH2. H3K27me3, histone H3 lysine 27 trimethylation; ChIP, chromatin immunoprecipitation.

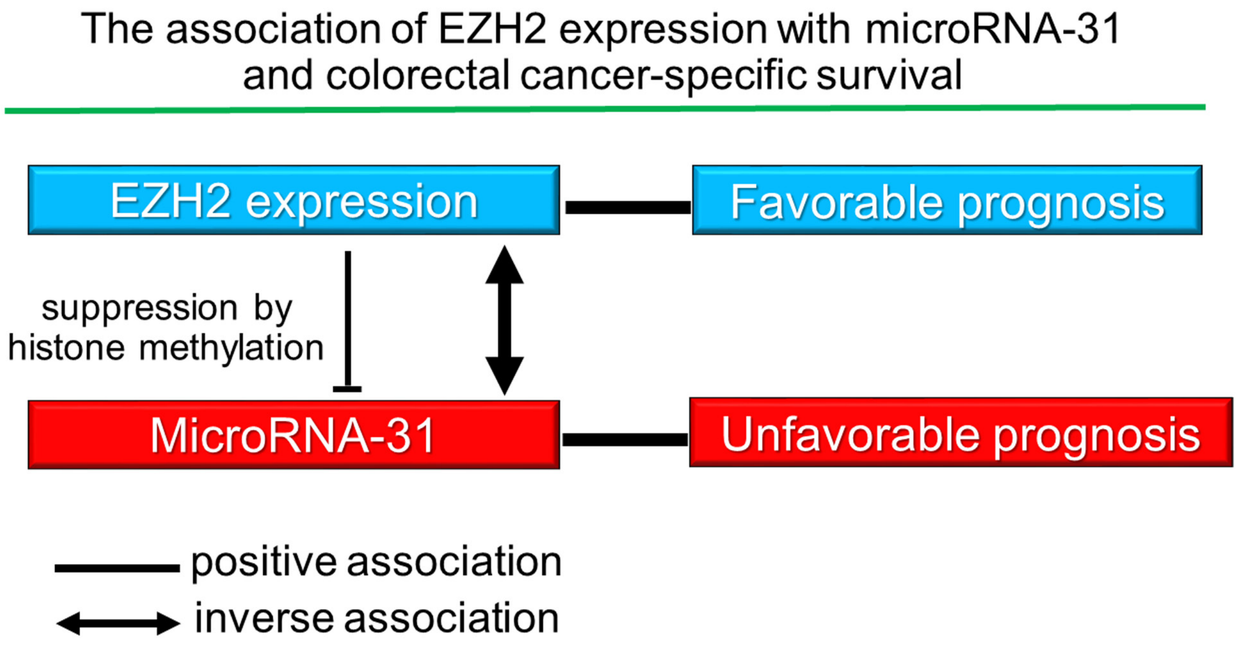

Supplementary Figure 6: The association of EZH2 expression with microRNA-31 and colorectal cancer-specific survival. Similar to previous studies, our current data showed that high EZH2 expression was significantly associated with favorable survival. These results are reasonable because we recently reported that high microRNA-31 expression, which is inversely correlated with EZH2 expression, was an unfavorable prognostic factor in patients with colorectal cancer.

Supplementary Table 1: Association of EZH2 expression with patient-mortality in colorectal cancers

EZH2 status	Total N	Cancer-specific survival		
		Univariate HR (95% CI)	Stage-stratified HR (95% CI)	Multivariate stage-stratified HR (95% CI)
Low expression group (score 0–2)	178	1 (referent)	1 (referent)	1 (referent)
High expression group (score 3)	123	0.48 (0.29-0.77)	0.51 (0.29-0.77)	0.46 (0.27-0.76)
P		0.0018	0.0022	0.0022

The multivariate, stage-stratified Cox model included the EZH2 expression variable stratified by gender, age at diagnosis, tumor size, year of diagnosis, tumor location, tumor differentiation, CIMP status, MSI status, mutations of *BRAF*, *KRAS*, and *PIK3CA*, and microRNA-31 expression.

CI, confidence interval; CIMP, CpG island methylator phenotype; HR, hazard ratio; MSI, microsatellite instability.

Supplementary Table 2: Sequences of chromatin immunoprecipitation (ChIP) primers

Name	Forward primer	Reverse primer
-1,000 bp	CCGATGACCTAGCCAGAAGT	CCCCACCCTTCAACTCGTAG
-500 bp	TATCCTCAACCCTCCGTGTC	CATACACCTGAAGGGGCAGT
+500 bp	CAATTTTGGCCCAGGAGATA	TTTCCGGGGACCTCTAGTTT
+850bp	TTTGATTCCGCCCTCTTTGG	AACGATGGCCCATGCATTTC
+42,500 bp	TGGCCTATTTGCTGTTCTAATGAC	GCAAGCCAACCCCAACA
+45,000 bp	AATGGGCCCTGCATTCTCT	AAAACCCACACCCTCACCAC
MYT	AGGCACCTTCTGTTGGCCGA	AGGCAGCTGCCTCCCGTACA
GAPDH	CGGCTACTAGCGGTTTTACG	AAGAAGATGCGGCTGACTGT
